# Validity, reliability, and invariance across sex of a German version of the Behavioral Regulation in Exercise Questionnaire

**DOI:** 10.3389/fpsyg.2024.1355928

**Published:** 2024-02-14

**Authors:** Armando Cocca, Martin Kopp, Klaus Greier, Karin Labek, Michaela Cocca, Gerhard Ruedl

**Affiliations:** ^1^Department of Human Movement Studies, University of Ostrava, Ostrava, Czechia; ^2^Department of Sport Science, University of Innsbruck, Innsbruck, Austria; ^3^Division of Physical Education, Private Educational College (KPH-ES), Stams, Austria; ^4^Institute of Psychology, University of Innsbruck, Innsbruck, Austria; ^5^Department of English Teaching Language, University of Ostrava, Ostrava, Czechia; ^6^Faculty of Economics and Management, Czech University of Life Sciences, Prague, Czechia

**Keywords:** motivation, self-determination theory, active behavior, validation, young adults

## Abstract

**Objective:**

Since there is no scientifically validated German version of the Behavioral Regulation in Exercise Questionnaire (BREQ-3), the aim of this study was to assess its psychometric parameters and invariance across sex in a sample of German-speaking young adults. The BREQ-3 is an instrument measuring the social and internal influences of motivation toward exercising. This tool is widespread within the scientific community and has been validated in several languages.

**Methods:**

A total of 271 participants (45% women; mean age = 20.67 ± 2.17 years; effect size ≥ 0.5) filled in the BREQ-3 at one time point, with a small sub-sample (*n* = 37) responding it a second time after 15 days. Confirmatory Factorial Analysis, Structural Modeling, and Intraclass Correlation Coefficient were used to examine the German version of the questionnaire.

**Results:**

Results highlighted a good fit of the six-dimensional model after the removal of two items (CFI = 0.912; SRMR = 0.0594; RMSEA = 0.064), as well as full invariance across sex (p_χ2_ = 0.218; ΔCFI < 0.01). Internal consistency and reliability were moderate to good.

**Conclusions:**

The 22-item German BREQ-3 is a scientifically valid instrument that can be used in cross-national studies dealing with social aspects of exercise behaviors.

## 1 Introduction

Motivation is one of the most prominent studied variables in human behavior and behavioral change (Deci and Ryan, 1985). According to the Self-Determination Theory (SDT; Deci and Ryan, 1985), motivation can be categorized in six different types along an internalization continuum representing the level of autonomy with which an individual will tend to carry out a behavior. One end of this continuum is represented by the most autonomous motivation to act (intrinsic motivation), and the opposite end by a total lack of drive (amotivation) (Center for Self-Determination Theory, 2022). Deci and Ryan (1985) provide a thorough description of each type of motivation in the continuum: the intrinsic one is the most internal one since it is activated by people's inner interests and enjoyment, and it is usually associated with the development of long-term habits; integrated motivation is the second most autonomy-guided type, since behaviors are driven by an individual's internal desire to be self-aware; a further step away from autonomy is represented by identified motivation, which describes behaviors as driven by personal values that an individual attributes to them, rather than enjoyment of carrying them out; introjected motivation is a more controlled type that is guided by an individual's need of self-control, which may depend on external sources, such as, for instance, fear of being judged by others; external motivation is the most externally controlled type in the continuum, since behaviors are regulated by fear of punishment for not carrying them out or by potential external rewards (for instance, receiving a gift for participating in an experiment); amotivation represents the final ending of the continuum, and it describes a person's complete lack of willingness to carry out a behavior (Center for Self-Determination Theory, 2022). Sport science is one among different scientific areas that have widely studied the interaction between motivation types and sources and behaviors, in particular in understanding what drives individuals to choose an active or sedentary lifestyle (Brandenbarg et al., 2023; Fang et al., 2023). This may have important consequences not only on the short term, but also on long-term health (Teixeira et al., 2012). Indeed, better exercise experience is associated with the intrinsic types of motivation (Liu et al., 2023). Other studies highlight that the most autonomous end of the continuum (intrinsic and integrated motivations) tend to have highly positive association with exercise-related parameters, whereas this association becomes negative as we move to the opposite end of the continuum (Durán-Vinagre et al., 2023; Fresno-Alba et al., 2023). For this reason, several scientific tools have been developed and tested over time to ensure a rigorous assessment of people's motivation in relation to sport and exercise (Plonczynski, 2000). In this sense, the Behavioral Regulation in Exercise Questionnaire (BREQ), based on the theoretical framework of SDT and developed by an exercise motivation research team from Bangor University, has become one of the most widely used instruments when the focus of research is active behavior related to health in the general population, as demonstrated by an extensive body of literature (e.g., Lev Arey et al., 2022; Mikkelsen et al., 2022; Vancampfort et al., 2023). The initial version of BREQ showed high levels of skewness for the “amotivation” items, which led to the exclusion of said subdomain, along with the “identified” one. However, Markland and Tobin (2004) were able to add “amotivation” in the second version of the instrument (BREQ-2), reporting good validity parameters. The BREQ-2 has now been translated and validated in several languages and has already been used successfully in scientific research worldwide. Nonetheless, despite constituting a sounder tool compared with the initial version, the BREQ-2 did not fully represent the motivation continuum as described in the SDT since it could not solve the issues with the “identified” subdomain. This was later addressed by Wilson et al. (2006), who were able to test a newer version of the questionnaire (BREQ-3) that included an extra item for the “introjected” subdomain along with a 4-item “identified” subdomain. The BREQ-3 reflects more accurately the six-motivation structure proposed in SDT's internalization continuum. The third version of the BREQ is composed by 24 items equally distributed in 6 sub-domains: amotivation (*I think exercising is a waste of time*); external regulation (*I exercise because other people say I should*); introjected regulation (*I feel ashamed when I miss an exercise session*); identified regulation (*I think it is important to make the effort to exercise regularly*); integrated regulation (*I exercise because it is consistent with my life goals*); and intrinsic regulation (*I find exercise a pleasurable activity*). Responses are given on a Likert scale ranging from 0 (not true for me) to 4 (very true for me). Average scores are used to establish the levels of each motivational regulation. The questionnaire has been validated in different languages, including Spanish (González-Cutre et al., 2010), Chinese (Luo et al., 2022), Italian (Cavicchiolo et al., 2022), Portuguese (Cid et al., 2018), or Malay (Chai et al., 2022), and extensively implemented in latest research focused on exercise and health (Chen et al., 2022; O'Loughlin et al., 2022; Sánchez-Herrera et al., 2022; Durán-Vinagre et al., 2023; Fresno-Alba et al., 2023; Lock et al., 2023; Reyes-Molina et al., 2023; etc.). A German version of the BREQ-3 translated by Rausch Osthoff (2017) is currently available online. However, although the BREQ-2 has already been validated by Witzki and Leyk (2014), the German version of BREQ-3 did not undergo any psychometric evaluation, hence, it cannot be considered scientifically valid in its current state and until a formal assessment of its psychometric parameters is provided. Despite its strength, its diffusion among the scientific community, and the fact that BREQ-3 allows to observe the entire internalization continuum and associated motivational sources, the lack of a scientifically validated version of the BREQ-3 in the German language represents a gap that needs to be filled. Indeed, providing a scientifically proven version of the German BREQ-3 would be an essential step not only for the research community in German-speaking countries, but it would also allow using a single, reliable tool in cross-national studies, with the possibility of comparing results of different communities and countries, finding common strategies for the promotion of active habits, as well as tailoring interventions based on regional differences. Therefore, the aim of this study was to test the validity of the German version of BREQ-3 provided by Rausch Osthoff (2017) in a population of young adults from Austria.

## 2 Materials and methods

### 2.1 Design

This is a validation study using quantitative, non-experimental and cross-sectional approach.

### 2.2 Sample

For confirmatory factor analysis by means of structural equation modeling, the minimum sample size necessary in order to achieve a large effect size (0.5) and statistical power (0.8) with a significance threshold set at 0.05 for a questionnaire composed by six latent variables and 24 items is 100 respondents (Westland, 2010). Our initial sample consisted of 298 young adults recruited from the population of first- and second-year bachelor students at the researchers' institution. Nonetheless, due to missing data (*n* = 21) or typos in the data transcription (*n* = 6), the final sample was composed by 271 respondents (122 women; mean age = 20.67 ± 2.17 years). For the reliability analysis, a smaller sample of 36 participants (mean age = 23.64 ± 1.93 years) responded to the questionnaire twice within 15 days (Streiner et al., 2015).

Formal approval from the Ethical Committee of the Institution had been previously provided. Signed informed consents were collected from all participants before the start of the data collection period.

### 2.3 Instruments

The BREQ-3 (Markland and Tobin, 2004; Wilson et al., 2006) is composed by 24 items distributed in six sub-domains (four items per sub-domain), as described above. Compared to the BREQ-2, this version includes an additional item in the “introjected regulation” sub-domain (item 22: *I would feel bad about myself if I was not making time to exercise*) and the “integrated regulation” sub-domain. Its translation to the German language was carried out and published online by Rausch Osthoff (2017). Since this author's version was adapted for sports training, their translation was maintained with the exception of the word “training,” which was substituted with “exercise,” thus reintroducing the actual meaning and focus by which the original BREQ-3 was created.

### 2.4 Data analysis

The questionnaire's psychometric parameters were tested using both IBM SPSS version 26 and IBM Amos version 22 software. Cronbach's Alpha and McDonald's Omega were calculated for the whole pool of items together and for each sub-domain separately in order to assess internal consistency. According to Hajjar (2018), internal consistency may be considered acceptable for values between 0.60 and 0.80, and good for values above 0.80. Regarding the structural assessment of BREQ-3, Confirmatory Factorial Analysis (CFA) with the Maximum Likelihood estimation method was run setting standardized estimates, residual moments, and modification indices as output for model fit evaluation (Schermelleh-Engel et al., 2003). Cut-off values for items' factor loadings were set at 0.50, whilst loading at or above 0.40 are considered sufficient (Fabrigar et al., 1999), whilst loading lower than 0.30 should be discarded (Field, 2013). In order to allow contrasting our parameters with those provided for the BREQ-3 in other languages, model fit assessment was carried out by examining the Comparative Fit Index (CFI; cut-off values at 0.90 or above); the Standardized Root Mean Square Residual (SRMR; cut-off values at 0.08 or below); and the Root Mean Square Error of Approximation (RMSEA; cut-off values at 0.08 or below) (Hu and Bentler, 1999; Cid et al., 2018). For poor model fit, the following criteria were used for model modifications: items with factor loading lower than 0.50 (sufficient) or below 0.40 (poor); and standardized residual covariances (SRC) between items, if higher than 2 (Fabrigar et al., 1999; Collier, 2020). Additionally, correlations among items within the same sub-scale and between each item and its sub-domain were carried out by means of Pearson's correlation analysis, with a significance level set at 0.05 or lower. This was done to monitor potential multicollinearity issues, with values above 0.70 considered at risk (Dormann et al., 2013), and for ensuring that the items and sub-scales correlated sufficiently and significantly, with item-item and item-sub-scale correlations recommended to be higher than 0.30 and higher than 0.50, respectively (Hajjar, 2018). Average Variance Extracted (AVE) and Composite Reliability (CR) were calculated for each dimension, as well. Recommended cut-off points are set at 0.50 for AVE (Fornell and Larcker, 1981), and at 0.70 for CR (Hair et al., 2014). These values have been used for testing each sub-scale convergent validity; discriminant validity is also established if the AVE of a sub-scale exceeds the squared correlation between that sub-scale and the others (Cid et al., 2018; Chai et al., 2022). Moreover, the Intraclass Correlation Coefficient, with a two-way mixed model and absolute agreement, was used to examine test-retest reliability of the instrument. According to Bobak et al. (2018), ICC values between 0.5 and 0.75 imply moderate reliability, whilst reliability is considered good for values between 0.75 and 0.9. Finally, configural and metric invariance based on sex was examined by testing an unconstrained model's fit and successively comparing it with a model with factor loadings constrained between males and females. As suggested by Putnick and Bornstein (2016), invariance is confirmed if the difference of Chi-square (χ^2^) between the two models is not significant (*p* > 0.05), and the absolute value of the CFI differential (ΔCFI) is lower than 0.01.

## 3 Results

**Figure 1:**
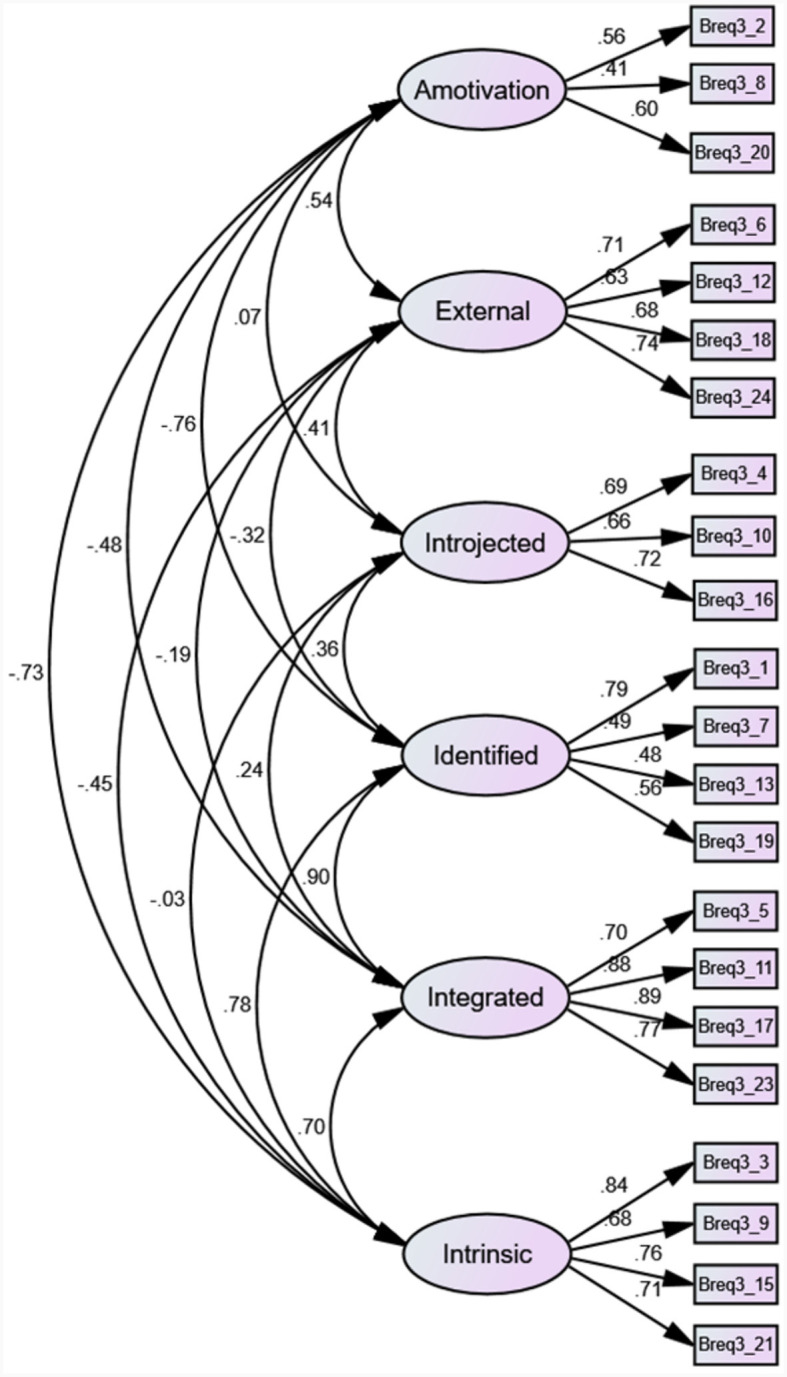
Structural model of the German Behavioral Regulation in Exercise (BREQ-3).

The 24-item version of the German BREQ-3 showed several issues during the first structural examination (χ^2^ = 593.035; df = 237; CFI = 0.877; SRMR = 0.0844; RMSEA = 0.073). High SRCs were found for the newly included item 22 (*I would feel bad about myself if I was not making time to exercise*), as well as for items 16 (*I feel like a failure when I haven't exercised in a while*), 8 (*I can't see why I should bother exercising*) and 7 (*I value the benefits of exercise*). Items 7 and 8, along with item 13 (*I think it is important to make the effort to exercise regularly*) had loading between 0.50 and 0.40, as well. The model was tested after the removal of each of these items individually, and an improved fit was found with the exclusion of item 22 (χ^2^ = 491.749; df = 215; CFI = 0.899; SRMR = 0.0632; RMSEA = 0.068). Nonetheless, the CFI was still below the acceptable threshold. Additionally, items 7, 14 (*I don't see the point in exercising*), and 20 (*I think exercising is a waste of time*) had too high SRCs. Loadings for items 7, 8, and 13 remained between 0.50 and 0.40. Again, the analysis of the structure was run after removing each of these items individually. The model further improved with the exclusion of item 14 (χ^2^ = 418.741; df = 194; CFI = 0.912; SRMR = 0.0594; RMSEA = 0.064), with all indexes indicating a good fit. The obtained model is shown in Figure 1.

Items' loadings in the final model ranged from 0.41 to 0.89. The correlational analysis among items within the same sub-scale delivered highly significant values (*p* < 0.001). No correlation coefficient surpassed the threshold set for multicollinearity (0.70). Item-sub-scale correlation coefficients ranged from 0.625 to 0.834 (*p* < 0.001). Internal consistency of the sub-scales is presented in Table 1.

**Table 1:** Internal consistency of the six sub-scales of the German Behavioral Regulation in Exercise Questionnaire (BREQ-3) and for the whole questionnaire.

**Sub-scale**	**Cronbach's Alpha**	**McDonald's Omega**
Amotivation (3 items)	0.602	0.607
External (4 items)	0.779	0.781
Introjected (3 items)	0.730	0.734
Identified (4 items)	0.652	0.662
Integrated (4 items)	0.880	0.884
Intrinsic (4 items)	0.839	0.842
BREQ-3 (22 items)	0.749	0.701

All sub-scales, as well as the BREQ-3 as a whole, obtained acceptable (>0.60) to good (>0.80) levels of internal consistency in the 22-item version presented above. Scores for AVE ranged from 0.51 to 0.74; for CR, values were between 0.76 and 0.91. The summary of AVE and CR for each sub-scale are provided in Table 2 below, along with sub-scale square correlations.

**Table 2:** Average Variance Extracted (AVE), Composite Reliability (CR), and square correlations between sub-scales of the final version of the German Behavioral Regulation in Exercise (BREQ-3).

**Sub-scale**	**CR**	**AVE**	**Square correlations**					
			**AM**	**EX**	**IJ**	**ID**	**IG**	**IM**
Amotivation	0.76	0.52	1	0.29	0.005	0.57	0.23	0.53
External	0.86	0.61	0.29	1	0.17	0.10	0.04	0.20
Introjected	0.85	0.65	0.005	0.17	1	0.13	0.06	0.001
Identified	0.81	0.51	0.57	0.10	0.13	1	0.81	0.61
Integrated	0.91	0.74	0.23	0.04	0.06	0.81	1	0.49
Intrinsic	0.89	0.68	0.53	0.20	0.001	0.61	0.49	1

AM, amotivation; EX, external; IJ, introjected; ID, identified; IG, integrated; IM, intrinsic.

Reliability was moderate for amotivation, external regulation, introjected regulation, and identified regulation (ICC values between 0.522 and 679), and good for integrated and intrinsic regulation (ICC = 0.789 and 0.752, respectively). The questionnaire as a whole showed good reliability (ICC = 0.773). A summary of the psychometric properties of the final German BREQ-3 contrasted with scores obtained in other translations is presented in Table 3.

**Table 3:** Psychometric parameters of the final version of the Behavioral Regulation in Exercise Questionnaire (BREQ-3) in different language translations.

	**CFI**	**SRMR**	**RMSEA**	**Factor loading range**	**Internal consistency range**
German version^*^	0.912	0.0594	0.064	0.41–0.89	α = 0.60–0.88 ω = 0.61–0.88
Chinese version (Luo et al., 2022)	0.98	NA	0.040	0.197–0.801	ω_c_ = 0.80–0.90
Italian version (Cavicchiolo et al., 2022)	0.96	0.040	0.050	0.51–0.95	ω = 0.65–0.94
Malay version (Chai et al., 2022)^†^	0.949	0.052	0.049	0.580–0.868	CR = 0.746–0.841
Portuguese version (Cid et al., 2018)	0.93	0.060	0.060	0.50–0.82	CR = 0.73–0.77
Spanish version (González-Cutre et al., 2010)	0.91	0.060	0.060	0.52–0.86	α = 0.66–0.87

^*^As presented in this paper;^†^the final model presents 5 factors; CR, composite reliability.

Sex invariance was tested by comparing the unconstrained 22-item model (configural invariance) with the model with constrained factor loadings (metric invariance). Both the model for males (*n* = 149) and the one for females (*n* = 122) showed parameters in the acceptable range (Table 4).

**Table 4:** Indexes of goodness of fit of the Behavioral Regulation in Exercise Questionnaire (BREQ-3) for the unconstrained and constrained model, and by sex.

**Model**	***χ^2^***	**df**	**CFI**	**SRMR**	**RMSEA**
Final 22-item model	418.741	194	0.912	0.0594	0.064
Unconstrained	636.724^†^	388	0.901^*^	0.0701	0.049
Constrained factor loadings	656.773^†^	404	0.900^*^	0.0729	0.049
Males	301.109	194	0.914	0.0706	0.061
Females	294.856	194	0.917	0.0719	0.064

^†^p = 0.218; ^*^ΔCFI = 0.001.

Chi-square comparison between unconstrained and constrained models was found to be not significant (*p* = 0.218). Additionally, the absolute value of ΔCFI was lower than the threshold of 0.01 (ΔCFI = 0.001).

## 4 Discussion

The aims of this study were to assess the psychometric parameters of a German version of the BREQ-3 and to examine its invariance by sex, in a sample of Austrian young adults.

The original model of the BREQ-3 (Markland and Tobin, 2004; Wilson et al., 2006) did not properly fit the data and participants of our study. However, the structure showed an acceptable fit after the removal of items 22 and 14. In particular, the inclusion of item 22 was one of the major changes that Wilson et al. (2006) implemented in BREQ-3 compared to its previous version, the BREQ-2. In our case, this new item seems to bring issues that affect the entire structure of the tool. Therefore, its removal led to reinstating the previously validated structure of the sub-scale of “introjected regulation” as presented in the BREQ-2, i.e., with three items. This constitutes no particular problem, considering that not only the structure of BREQ-2 (including the mentioned sub-scale) had been already validated both in its original language (Markland and Tobin, 2004) and in German (Witzki and Leyk, 2014), but it was also widely used in previous literature in the field of exercise and health (Jekauc et al., 2021; Kovács and Kovács, 2021; Ostendorf et al., 2021). Regarding item 14, which belonged to the “amotivation” sub-scale, our findings are not in line with the outcomes from validation processes in other languages (González-Cutre et al., 2010; Cid et al., 2018; Cavicchiolo et al., 2022; Chai et al., 2022; Luo et al., 2022). Nonetheless, none of these processes was able to confirm the original 24-item model, which, with some differences, always delivered a poor fit in its initial form. For instance, González-Cutre et al. (2010) obtained a proper fit for the Spanish BREQ-3 only after removing one item from the “identified regulation” sub-scale. On the other hand, Chai et al. (2022) validated the BREQ-3 with a 5-sub-scale structure, and i.e., they were forced to remove an entire sub-scale to obtain a sound model in a sample of Malay young adults. The Portuguese version of BREQ-3 required the elimination of one item per each sub-scale in order to obtain a fitting model, and consequently, that version of the BREQ-3 was confirmed with a total of 18 items equally distributed into the six original sub-scales (Cid et al., 2018). These results led some authors to directly translate Cid's 18-item version of the questionnaire, rather than the original one (Cavicchiolo et al., 2022). Issues were also found in validating the Chinese version of the 24-item BREQ-3 (Luo et al., 2022), with the authors suggesting that the original structure proposed by Markland and Tobin (2004) might need to be revised. In this sense, the elimination of items from our version seems in line with the procedure carried out for the existing models in other languages. The fact that each of these models differs in which particular items from the original model were controversial may be attributed to regional differences requiring cultural adaptation (Huang and Wong, 2014).

Regardless of the above-mentioned cultural differences, the parameters found in our final version of the instrument are in line with those reported in other adaptations. In fact, compared to the CFI for the German version (CFI = 0.91), values for the other versions ranged from 0.91 to 0.94, except the Italian (CFI = 0.96) and the Chinese one (CFI = 0.98) reporting values above 0.95 (Cavicchiolo et al., 2022; Luo et al., 2022). Similarly, SRMR for other adaptations also remained usually in the range of 0.05 to 0.07 and was 0.059 in our study. The RMSEA was perhaps the index with the greatest fluctuation, with the poorest value (RMSEA = 0.09) reported in the original English version (Wilson et al., 2006), whilst the best (RMSEA = 0.04) in the Chinese ones (Luo et al., 2022). In our case, an RMSEA of 0.064 represents an average value compared to those previously reported. Test-retest reliability was only reported for the Spanish adaptation (González-Cutre et al., 2010), and only for the questionnaire as a whole. Although their reported ICC was higher (0.90) than in our study, both remain within the range considered as good.

Finally, our outcomes indicate full invariance for the 22-item German BREQ-3 by sex, the questionnaire needing no further modifications depending on the sex of the participants. This is in line with previous studies, also reporting full invariance by sex for their BREQ-3 versions (González-Cutre et al., 2010; Cid et al., 2018; Cavicchiolo et al., 2022; Luo et al., 2022).

### 4.1 Limitations

The main limitation of this study is represented by the lack of other valuable validation procedures, i.e., construct and criterion-related validity. These procedures tend to play a more essential role in untested or newly created questionnaires, which is not the case with the BREQ-3. Although we calculated convergent and discriminant validity by means of AVE and CR scores, following the same procedure as other country-specific BREQ-3 validation studies (Cid et al., 2018; Chai et al., 2022), studying these parameters using comparable gold-standard tools provided in the literature would bring further strength to this work. Additionally, not all valid measurements for the mentioned procedures are available in German, meaning that additional steps would be required. Nonetheless, they always provide additional information and may help to further confirm the soundness of the model proposed in this work. Another limitation may be that the model presented in this work was tested with a population of young adults only. Considering that the original instrument has been used in different environments and with different age ranges, including youth and elderly, an important step forward would be to assess measurement invariance by age, as well as to test the instrument in special populations (sedentary, different types of diseases, etc.). An additional recommendation would be to use the Basic Psychological Need Satisfaction Scale (BPNSS; Deci and Ryan, 2000) for construct convergent validity - currently, there is no German version of this tool -, since this questionnaire is built within the same SDT framework and it has been already used with such purpose (Luo et al., 2022); and to gather information on weekly physical activity (self-reported or via accelerometry) for criterion-related predictive validity, as it is known that different types of motivation are associated with exercise habits (Kalajas-Tilga et al., 2022).

## 5 Conclusions

As could have been expected based on the results of other language adaptations, the German version of the BREQ-3 could not be confirmed in its original 24-item structure. Nonetheless, the final 22-item version presented in this study shows good indexes of goodness of fit and full invariance across sex, at the same time as it maintains the main feature that made the original BREQ-3 widespread within the scientific community, i.e., the inclusion of all six types of motivation as described in the framework of the SDT. Although further examination is required to verify its structural stability across ages and populations, the German version of the BREQ-3 proposed in this study is scientifically robust and may be recommended to be used in future research in the field of sport sciences and, in particular, exercise and health.

## Data availability statement

The raw data supporting the conclusions of this article will be made available by the authors, without undue reservation.

## Ethics statement

The studies involving humans were approved by Board for Ethical Questions in Science of the University of Innsbruck. The studies were conducted in accordance with the local legislation and institutional requirements. The participants provided their written informed consent to participate in this study.

## Author contributions

AC: Data curation, Formal analysis, Methodology, Writing — original draft. MK: Methodology, Resources, Visualization, Writing — review & editing. KG: Conceptualization, Investigation, Resources, Writing — review & editing. KL: Validation, Writing — review & editing. MC: Formal analysis, Methodology, Writing — review & editing. GR: Conceptualization, Investigation, Supervision, Writing — review & editing.

## References

[B1] Bobak C. A. Barr P. J. O'Malley A. J. (2018). Estimation of an inter-rater intra-class correlation coefficient that overcomes common assumption violations in the assessment of health measurement scales. BMC Med. Res. Methodol. 18, 93. 10.1186/s12874-018-0550-6 30208858 PMC6134634

[B2] Brandenbarg P. Krops L. A. Seves B. L. Hoekstra T. Hettinga F. J. Twisk J. W. R.. (2023). Psychosocial factors of physical activity among people with disabilities: prospective cohort study. Rehabil. Psychol. 68,164–173. 10.1037/rep0000488 36780269

[B3] Cavicchiolo E. Sibilio M. Lucidi F. Cozzolino M. Chirico A. Girelli L.. (2022). The psychometric properties of the behavioural regulation in Exercise Questionnaire (BREQ-3): factorial structure, invariance and validity in the Italian context. Int. J. Environ. Res. Public Health 19:1937. 10.3390/ijerph19041937 35206126 PMC8872217

[B4] Center for Self-Determination Theory (2022). Theory Overview. Available online at: https://selfdeterminationtheory.org/theory/ (accessed June 21, 2023).

[B5] Chai S. Kueh Y. C. Majdi Yaacob N. Kuan G. (2022). Psychometric properties of the Malay version of the Behavioural Regulation in Exercise Questionnaire (BREQ-3). PLoS ONE 17:e0269099. 10.1371/journal.pone.0269099 35749451 PMC9231722

[B6] Chen X. Yang S. Zhao H. Li R. Luo W. Zhang X. (2022). Self-efficacy, exercise anticipation and physical activity in elderly: using Bayesian networks to elucidate complex relationships. Patient Prefer. Adherence 16, 1819–1829. 10.2147/PPA.S369380 35923659 PMC9342886

[B7] Cid L. Monteiro D. Teixeira D. Teques P. Alves S. Moutão J.. (2018). The behavioral regulation in exercise questionnaire (BREQ-3) Portuguese-version: evidence of reliability, validity and invariance across gender. Front. Psychol. 9:1940. 10.3389/fpsyg.2018.01940 30364299 PMC6193427

[B8] Collier J. E. (2020). Applied Structural Equation Modeling Using AMOS: Basic to Advanced Techniques. New York, NY: Routledge.

[B9] Deci E. L. Ryan R. M. (1985). Intrinsic Motivation and Self-Determination in Human Behavior. New York, NY: Plenum.

[B10] Deci E. L. Ryan R. M. (2000). The “what” and “why” of goal pursuits: human needs and the self-determination of behavior. Psychol. Inq. 11, 227–268. 10.1207/S15327965PLI1104_01

[B11] Dormann C. F. Elith J. Bacher S. Buchmann C. Carl G. Carré G.. (2013). Collinearity: a review of methods to deal with it and a simulation study evaluating their performance. Ecography 36, 27–46. 10.1111/j.1600-0587.2012.07348.x

[B12] Durán-Vinagre M. Á. Ibáñez S. J. Feu S. Sánchez-Herrera S. (2023). Analysis of the motivational processes involved in university physical activity. Front. Psychol. 13:1080162. 10.3389/fpsyg.2022.1080162 36698566 PMC9868708

[B13] Fabrigar L. R. Wegener D. T. MacCallum R. C. Strahan E. J. (1999). Evaluating the use of exploratory factor analysis in psychological research. Psychol. Methods 4, 272–299.

[B14] Fang C.-Y. Chen P.-Y. Liao Y. (2023). Factors influencing seniors' willingness to pay intention for exercise in the civil sports and recreation centers. Front Public Health 10:992500. 10.3389/fpubh.2022.992500 36777771 PMC9911538

[B15] Field A. (2013). Discovering Statistics using SPSS, 4th edn. London: SAGE.

[B16] Fornell C. Larcker D. F. (1981). Evaluating structural equation models with unobservable variables and measurement error. J. Mark. Res 18, 39–50. 10.2307/3151312

[B17] Fresno-Alba S. Leyton-Román M. Mesquita da Silva S. Jiménez-Castuera R. (2023). Predicting quality of life in women with breast cancer who engage in physical exercise: the role of psychological variables. Healthcare 11:2088. 10.3390/healthcare11142088 37510529 PMC10379105

[B18] González-Cutre D. Sicilia Á. Fernández A. (2010). Hacia una mayor comprensión de la motivación en el ejercicio físico: medición de la regulación integrada en el contexto español. Psicothema 22, 841–847. 21044522

[B19] Hair J. F. Hult G. T. M. Ringle C. M. Sarstedt M. (2014). A Primer on Partial Least Squares Structural Equation Modelling (PLS-SEM). Los Angeles, CA: SAGE Publications.

[B20] Hajjar S. (2018). Statistical analysis: internal-consistency reliability and construct validity. Int. J. Quant. Qual. Res. Method. 6, 46–57

[B21] Hu L. T. Bentler P. M. (1999). Cutoff criteria for fit indexes in covariance structure analysis: conventional criteria versus new alter-natives. Struct. Equ. Modeling 6, 1–55. 10.1080/10705519909540118

[B22] Huang W. Y. Wong S. H. (2014). “Cross-cultural validation”, in Encyclopedia of Quality of Life and Well-Being Research (Dordrecht, NL: Springer Dordrecht), 1369–1371. 10.1007/978-94-007-0753-5_630

[B23] Jekauc D. Rayling S. Klopp S. Schmidt D. Rittmann L. -M. Fritsch J. (2021). Effects of a web-based rehabilitation aftercare on subjective health, work ability and motivation: a partially randomized controlled trial. BMC Musculoskelet. Disord. 22, 366. 10.1186/s12891-021-04239-z 33874917 PMC8054846

[B24] Kalajas-Tilga H. Hein V. Koka A. Tilga H. Raudsepp L. Hagger M. S. (2022). Application of the trans-contextual model to predict change in leisure time physical activity. Psychol. Health 37, 62–86. 10.1080/08870446.2020.1869741 33405970

[B25] Kovács K. Kovács K. E. (2021). Using the behavioural regulation in an Exercise Questionnaire (BREQ-2) in central and eastern Europe: evidence of reliability, sociocultural background, and the effect on sports activity. Int. J. Environ. Res. Public Health 18:11834. 10.3390/ijerph182211834 34831587 PMC8619575

[B26] Lev Arey D. Blatt A. Gutman T. (2022). A self-determination theory and acceptance and commitment therapy-based intervention aimed at increasing adherence to physical activity. Front. Psychol. 13:935702. 10.3389/fpsyg.2022.935702 36051214 PMC9426339

[B27] Liu J. Ullrich-French S. Qiu Y. Mao Z. X. (2023). An exploratory study: profiles of trait mindfulness and associations with intrinsic motivation and affective exercise experiences. Mindfulness 14, 2975–2987. 10.1007/s12671-023-02255-9

[B28] Lock M. Post D. Dollman J. Parfitt G. (2023). The effects of a theory-informed intervention on physical activity behaviour, motivation and well-being of frontline aged care workers: a pilot study with 6-month follow-up. Health Promot. J. Austr. 35, 207–219. 10.1002/hpja.740 37158108

[B29] Luo Y. Mullin E. M. Mellano K. T. Sha Y. Wang C. (2022). Examining the psychometric properties of the Chinese behavioral regulation in exercise questionnaire-3: a bi-factor approach. PLoS ONE 17:e0265004. 10.1371/journal.pone.0265004 35255098 PMC8901058

[B30] Markland D. Tobin V. (2004). A modification of the behavioral regulation in exercise questionnaire to include an assessment of amotivation. J. Sport Exerc. Psychol. 26, 191–196. 10.1123/jsep.26.2.191

[B31] Mikkelsen N. Dall C. H. Frederiksen M. Holdgaard A. Rasmusen H. Prescott E. (2022). The motivation for physical activity is a predictor of VO2peak and is a useful parameter when determining the need for cardiac rehabilitation in an elderly cardiac population. PLoS ONE 17:e0275091. 10.1371/journal.pone.0275091 36170331 PMC9518852

[B32] O'Loughlin E. K. Riglea T. Sylvestre M.-P. Pelekanakis A. Sabiston C. M. Bélanger M.. (2022). Stable physical activity patterns predominate in a longitudinal study of physical activity among young adults in Canada from before to during the COVID-19 pandemic. Prev. Med. Rep. 27:101782. 10.1016/j.pmedr.2022.101782 35392180 PMC8980605

[B33] Ostendorf D. M. Schmiege S. J. Conroy D. E. Phelan S. Bryan A. D. Catenacci V. A. (2021). Motivational profiles and change in physical activity during a weight loss intervention: a secondary data analysis. Int. J. Behav. Nutr. Phys. Act. 18, 158. 10.1186/s12966-021-01225-5 34863198 PMC8642857

[B34] Plonczynski D. J. (2000). Measurement of motivation for exercise. Health Educ. Res. 15, 695–705. 10.1093/her/15.6.695 11142077

[B35] Putnick D. L. Bornstein M. H. (2016). Measurement invariance conventions and reporting: the state of the art and future directions for psychological research. Dev. Rev. 41, 71–90. 10.1016/j.dr.2016.06.004 27942093 PMC5145197

[B36] Rausch Osthoff A.-K. (2017). Behavioural Regulation in Exercise Questionnaire (BREQ-3) - Deutsche Version. ZHAW Digital Collection.35749451

[B37] Reyes-Molina D. Nazar G. Cigarroa I. Carrasco Marín F. Cárcamo Regla R. Rozas Pardo K.. (2023). Motivation, barriers and benefits for the practice of physical exercise in a mobile health intervention in adults from Biobío, Chile). Retos 49, 623–631. 10.47197/retos.v49.97141

[B38] Sánchez-Herrera S. Cubero J. Feu S. Durán-Vinagre M. Á. (2022). Motivation regarding physical exercise among health science university students. Int. J. Environ. Res. Public Health 19:6524. 10.3390/ijerph19116524 35682107 PMC9180503

[B39] Schermelleh-Engel K. Moosbrugger H. Müller H. (2003). Evaluating the fit of structural equation models: tests of significance and descriptive goodness-of-fit measures. Methods Psychol Res 8, 23–74.

[B40] Streiner D. Norman G. Cairney J. (2015). Health Measurement Scales. A Practical Guide to Their Development and Use, 5th edn. Oxford: Oxford University Press.

[B41] Teixeira P. J. Carraça E. V. Markland D. Silva M. N. Ryan R. M. (2012). Exercise, physical activity, and self-determination theory: a systematic review. Int. J. Behav. Nutr. Phys. Act. 9:78. 10.1186/1479-5868-9-78 22726453 PMC3441783

[B42] Vancampfort D. De Soir E. Ramos-Sanchez C. P. van Winkel R. Louw Q. A. McKeon G.. (2023). Autonomous motivation for exercise is key to an active lifestyle in firefighters. Workplace Health Saf. 71, 238–244. 10.1177/21650799221147174 36695171

[B43] Westland J. C. (2010). Lower bounds on sample size in structural equation modeling. Electron. Commer. Res. Appl. 9, 476–487. 10.1016/j.elerap.2010.07.003

[B44] Wilson P. M. Rodgers W. M. Loitz C. C. Scime G. (2006). “It's who I am…really!” the importance of integrated regulation in exercise contexts. J. Biobehav. Res. 11, 79–104. 10.1111/j.1751-9861.2006.tb00021.x

[B45] Witzki A. Leyk D. (2014). “Behavioral Regulation in Exercise Questionnaire (BREQ-2): reliability and validity of a German translation”, in Supplement to Psychological Test and Assessment Modeling, ed. O. Güntürkün (Lengerich: Pabst Science Publisher), 427.

